# AANG: A natural compound formula for overcoming multidrug resistance via synergistic rebalancing the TGF‐β/Smad signalling in hepatocellular carcinoma

**DOI:** 10.1111/jcmm.16928

**Published:** 2021-09-12

**Authors:** Jeff Yat‐Fai Chung, Max Kam‐Kwan Chan, Philip Chiu‐Tsun Tang, Alex Siu‐Wing Chan, Justin Shing‐Yin Chung, Xiao‐Ming Meng, Ka‐Fai To, Hui‐Yao Lan, Kam‐Tong Leung, Patrick Ming‐Kuen Tang

**Affiliations:** ^1^ Department of Anatomical and Cellular Pathology State Key Laboratory of Translational Oncology The Chinese University of Hong Kong Shatin Hong Kong; ^2^ Department of Applied Social Sciences The Hong Kong Polytechnic University Kowloon Hong Kong; ^3^ School of Pharmacy Anhui Medical University Hefei China; ^4^ Department of Medicine and Therapeutics Li Ka Shing Institute of Health Sciences The Chinese University of Hong Kong Shatin Hong Kong; ^5^ Department of Paediatrics Prince of Wales Hospital The Chinese University of Hong Kong Shatin Hong Kong

**Keywords:** AANG, Asiatic acid, hepatocellular carcinoma, multidrug resistance, naringenin, p‐glycoprotein, TGF‐β/Smad signalling

## Abstract

Cancer cells are high in heterogeneity and versatility, which can easily adapt to the external stresses via both primary and secondary resistance. Targeting of tumour microenvironment (TME) is a new approach and an ideal therapeutic strategy especially for the multidrug resistant cancer. Recently, we invented AANG, a natural compound formula containing traditional Chinese medicine (TCM) derived Smad3 inhibitor Naringenin (NG) and Smad7 activator Asiatic Acid (AA), for rebalancing TGF‐β/Smad signalling in the TME, and its implication on the multidrug resistance is still unexplored. Here, we observed that an equilibrium shift of the Smad signalling in patients with hepatocellular carcinoma (HCC), which was dramatically enhanced in the recurrent cases showing p‐glycoprotein overexpression. We optimized the formula ratio and dosage of AANG that effectively inhibit the proliferation of our unique human multidrug resistant subclone R‐HepG2. Mechanistically, we found that AANG not only inhibits Smad3 at post‐transcriptional level, but also upregulates Smad7 at transcriptional level in a synergistic manner in vitro. More importantly, AANG markedly suppressed the growth and p‐glycoprotein expression of R‐HepG2 xenografts in vivo. Thus, AANG may represent a novel and safe TCM‐derived natural compound formula for overcoming HCC with p‐glycoprotein‐mediated multidrug resistance.

## 1 INTRODUCTION

Cancer is still the top leading cause of death in Hong Kong and worldwide, but treatment remains ineffective with severe side effects. This may largely due to the heterogeneity of cancers and the development of secondary drug resistance. Increasing evidence shows that growth, invasion, metastasis and multidrug resistance are largely relied on the tumour microenvironments (TME)^1^, ^2^; suggesting therapeutics that can target protumoural microenvironments may represent as an effective approach for overcoming multidrug resistant cancer.

Increasing evidence shows transforming growth factor‐β1 (TGF‐β1) is responsible for TME formation.^3^, ^4^, ^5^ Interestingly, TGF‐β1 is suggested to suppress carcinogenesis, but paradoxically supports cancer progression once TME has established.^6^, ^7^, ^8^, ^9^ For example, cancer cell‐derived TGF‐β1 silences the host anti‐tumour immunity by inducing regulatory T (Treg) cells,^4^ which results in cancer evasion from the host immune surveillance. TGF‐β1 also acts as a cancer promoter by enhancing angiogenesis, epithelial‐mesenchymal transition (EMT) and extracellular matrix (ECM) degradation in TME.^6^, ^7^, ^8^, ^9^ In hepatocellular carcinoma (HCC), TGF‐β alters TGF‐β receptors expression, Smads activation and induction to initiate oncogenes transcription for promoting cancer cell motility and EMT.^10^ In addition, TGF‐β1 is reported to increase drug resistance via p‐glycoprotein expression in cancer cells.^11^, ^12^ TGF‐β1 signalling is a major pathway for promoting the progression of inflammatory diseases,^1^, ^13^, ^14^, ^15^, ^16^ where Smad3 is the key pathogenic mediator by regulating disease development at transcriptional level.^16^, ^17^, ^18^, ^19^, ^20^, ^21^ Indeed, Smad3‐deficient mice were resistant to chemical‐induced skin carcinogenesis,^22^, ^23^ and our group further demonstrated the important role of Smad3‐dependent TME (Smad3‐TME) in cancer progression on two syngeneic mouse cancer models bearing invasive lung carcinoma LLC and melanoma B16F10.^21^

We have developed natural compound formula AANG, which is derived from traditional Chinese medicine (TCM) and effectively inhibited Smad3‐dependent cancer progression in mice via suppressing Smad3 but reactivating Smad7 in the TME.^24^ Naringenin (NG) is a predominant flavanone isolated from Millettia reticulata Benth with pharmacology properties such as anticancer, antimutagenic, anti‐inflammatory and antiatherogenic activities.^25^ The cancer‐inhibitory effects of NG have been linked to the regulation of various signalling pathways, such as Nrf2, NF‐κB, PI3K/Akt/mTOR, Jnk, Erk and p38 MAPK.^26^, ^27^ It also intervenes with the function of various signalling molecules, such as caspases, Bax, TNF‐α, Bcl‐2 and VEGF.^28^ Asiatic acid (AA) is a triterpenoid component isolated from Centella asiatica, functions as a Smad7 agonist shows pharmacological effects on anti‐inflammation, antioxidation, anti‐tumour, neuroprotection, hepatoprotection and wound healing.^29^ AA largely suppressed cancer cell proliferation and survival by regulating multiple pathways through direct and indirect interactions, including the downregulation of NF‐κB, the suppression of AP‐1 activity and various effects on the STAT proteins.^30^ Nevertheless, the therapeutic potential of AANG in cancer with secondary drug resistance is still unexplored.

In this study, we evaluated this novel TCM‐derived natural compound formula on human primary and recurrent hepatocellular carcinoma (HCC). The specificity, therapeutic dosage, anticancer efficacy and the safety of AANG were intensively defined by using our unique multidrug resistant HCC cell line R‐HepG2^31^ in vitro and in vivo. This work provided important rationales for further developing AANG as a novel therapeutic strategy for overcoming hepatocarcinoma with P‐glycoprotein mediated multidrug resistance.

## 2 MATERIALS AND METHODS

### 2.1 Patient samples

Archival formalin‐fixed paraffin‐embedded (FFPE) tissue specimens were retrieved from patients with hepatocarcinoma aged between 50 and 70 who underwent colectomy at the Prince of Wales Hospital, Hong Kong SAR. The study was conducted according to the principles expressed in the Declaration of Helsinki. Written informed consent was obtained from all the patients. This study was approved by the Clinical Research Ethics Committee of Joint Chinese University of Hong Kong‐New Territories East Cluster (Ref. No. NTEC‐2017‐0546).

### 2.2 Animals and treatments

Nude (8‐ to 10‐week‐old) mice were purchased from the Chinese University of Hong Kong Laboratory Animal Services Centre. All experimental procedures were approved by the Animal Ethics Experimental Committee of the Chinese University of Hong Kong.

Two Traditional Chinese Medicine (TCM)‐based drugs were used in this study, including AA (97% HPLC purified, Sigma‐Aldrich 464‐92‐6) and NG (98% HPLC purified, Sigma‐Aldrich 67604‐48‐2). Both drugs were dissolved in the DMSO as a solvent. Tumour‐bearing mice were randomly divided into groups for control or treatment with AANG at a combined dose with AA (25 mg/kg) and NG (25 mg/kg) daily via intraperitoneal injection.

### 2.3 Cell culture

HepG2 cells (ATCC HB‐8065) were selected with increasing concentrations of Doxorubicin (Dox, Selleckchem) from 0.1 to 100 μM during cell passages. After several rounds of selection, R‐HepG2 with MDR properties was obtained and then cultured with 1.2 μM Dox to maintain its drug resistance.^31^ R‐HepG2 human cancer cells were then cultured in DMEM (Life Technologies), respectively, with 10% heat‐inactivated FBS (Life Technologies), 1% penicillin and streptomycin (Life Technologies) in 5% CO_2_ at 37°C. For in vitro assays, the R‐HepG2 cells were treated with AA, NG or TGF‐β1 (Gibco PHG9204). Cell lines were free of mycoplasma by cultured with antimicrobial reagent Normocin (InvivoGen) 2 weeks prior to experiments.

### 2.4 Histology and Immunofluorescence Staining

Mice organ sections were fixed in 4% paraformaldehyde, stained with the haematoxylin‐eosin (H&E staining). Human liver and hepatocellular carcinoma tissues were performed on 5 μm FFPE sections and stained with the antibody against p‐Smad3, Smad7, TGF‐β1 and MDR1 (1:1000, Santa Cruz Biotechnology, Table S1). The protein expression on the TMA slides was calculated based on the histoscore (H‐score) method. Samples were imaged on the Ni‐u Light Microscope (Nikon) and analysed by Aperio ImageScope (Leica Biosystems).

Immunofluorescence was performed on 5 μm fresh tissue sections from human tumours, mouse tumours and spleen tissues and stained with antibodies against p‐smad3, Smad7 and MDR1 performed as previously described.^24^ Antibodies were diluted to be 1:100 in staining buffer (eBioscience 00‐4222‐57) and applied on the samples at 4°C for overnight. The unbounded antibodies were washed out with PBST 3 times followed by detection with Alexa 488‐conjugated secondary antibodies (Life Technologies). The samples were sealed with DAPI mounting buffer (Invitrogen S36938). All stained samples were imaged under a fluorescence microscope (Axio Observer.Z1; Carl Zeiss^19^).

### 2.5 MTT assay

The MTT assay was used to determine the cytotoxicity of AANG on R‐HepG2 cells in vitro. In brief, R‐HepG2 cells (1 × 10^4^ cells/well) were seeded on a 96‐well plate and serial dilutions of AA, NG or their combination with indicated concentration were added on the next day. After 24‐h of treatment, 30 microlitres of methyl‐thiazoldiphenyl tetrazolium (MTT) (5 mg/ml) was added to each well and incubated for 2 h at 37°C. The MTT solution was then replaced by 100 µl of dimethyl sulphoxide in each well and measured with a microtitre‐plate reader (Bio‐Rad) at 540 nm, and all data were calculated as percentage against the control.

### 2.6 Western blot analysis

Proteins in mouse tumour tissue were extracted by chilled radioimmunoprecipitation assay lysis buffer (RIPA, Pierce) and then examined by Western blot analysis with primary antibodies against, p‐Smad3, Smad3, Smad7 (all at 1:1000 dilution) and glyceraldehyde 3‐phosphate dehydrogenase (GAPDH) (1:10,000 dilution, Table S1), followed by incubation with the corresponding IRDyeTM800‐conjugated secondary antibodies (1:10,000, Rockland Immunochemical) performed as previously described.^23^ GAPDH was used as an internal control. Expression levels of the proteins were detected by using the LiCor/Odyssey infrared image system (LI‐COR; Biosciences), and the band intensities were quantified with ImageJ software (version 1.48, NIH).

### 2.7 real‐time PCR

Total RNA from the R‐HepG2 cells was isolated by using TRIzol reagent (Life Technologies) according to the manufacturer's instructions and quantified by using ND‐2000 Nanodrop (Thermo Scientific). Total RNA (1 μg) was used to synthesize the first strand of cDNA as described previously. Relative mRNA expression was measured using the iQTM SYBR Green Supremix on Opticon2 system (Bio‐Rad). The primers used in this study, including mouse Smad3, Smad7 and GAPDH, have been previously described.^32^, ^33^ The primers used in this study included MDR1 forward 5′‐GTCGTGATGGAACTTGAA‐3′ and reverse 5′‐GCTTTCTGTGGACACTTCTG‐3′. The relative expression levels of target genes were normalized with GAPDH and calculated using the 2^−ΔΔCt^ method.

### 2.8 Enzyme‐linked immunosorbent assay

Serum samples from tumour‐bearing mice were collected to detect cytotoxicity indicators using the enzyme‐linked immunosorbent assay (ELISA) kit as previously described.^21^ ALT (TR71121, Thermo scientific), AST (TR70121, Thermo scientific), LDH (J2380, Promega) and creatinine (ab65340, Abcam) were measured according to the instructions of the manufacturer.

### 2.9 Statistical analysis

Statistical analysis was performed in GraphPad Prism 5 (GraphPad Software). All data were presented as mean ± SEM. Statistical significance was determined by *p* < 0.05 in the standard *t* test or one‐way or two‐way ANOVA.

## 3 RESULTS

### 3.1 Equilibrium shift of Smad signalling in recurrent HCC

**FIGURE 1:**
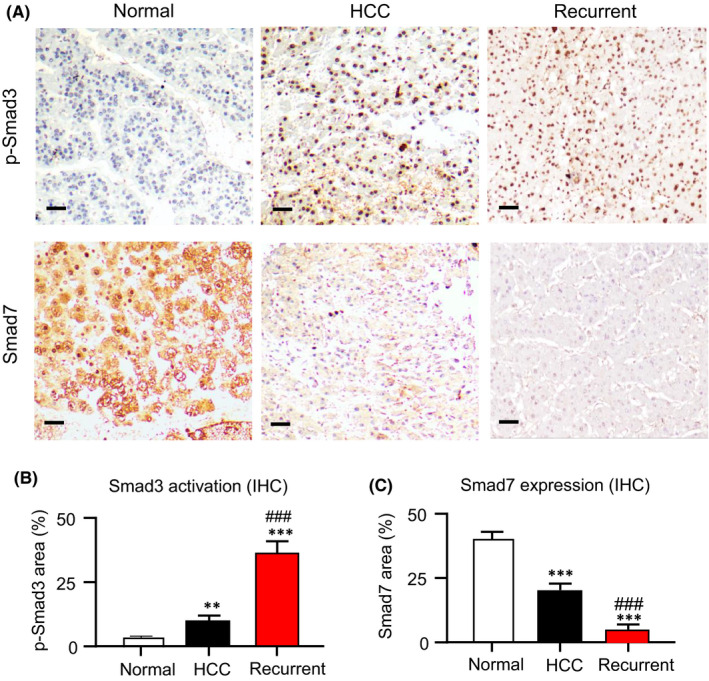
Imbalance of Smads signalling in primary and recurrent hepatocellular carcinoma (HCC) patients. Equilibrium shift of Smads signalling was detected in both primary and recurrent HCC. (A) Immunohistochemistry revealed the activation of Smad3 but suppression of Smad7 in primary HCC compared to the normal liver, which was further enhanced in the recurrent cases showing by the quantifications of (B) Smad3 activation and (C) Smad7 expression. Results are representative images of 5 biopsies. **p < 0.01, ***p < 0.001 vs. Normal, ^###^ *p* < 0.001 vs. HCC group, *n* = 5, one‐way ANOVA. Scale bars (A) 50 μm

As Smad signalling is important for cancer progression,^5^, ^21^ but its contribution in HCC is still largely unclear, especially on the recurrent cases. Therefore, we investigated the equilibrium of Smad signalling in both primary and recurrent HCC via immunohistochemistry assay. In line with our notion, we detected a hyperactivation of Smad3 but inhibition of Smad7 in the primary HCC compared to the paired non‐tumour liver tissue (Figure 1A). Interestingly, a dramatic equilibrium shift of Smad3/Smad7 signalling was observed in the recurrent cases compared to the biopsies of primary HCC and normal liver (Figure 1B,C), highlighting the importance of Smad signalling in HCC progression. More surprisingly, we unexpectedly found the induction of multidrug resistance gene MDR1, p‐glycoprotein, is strongly associated with the imbalance of Smad3/Smad7 signalling in both the primary and recurrent HCC (Figure S1), implying a potential role of Smad signalling in the development of HCC multidrug resistance.

### 3.2 AANG synergistically inhibits multidrug resistant HCC cells in vitro

**FIGURE 2:**
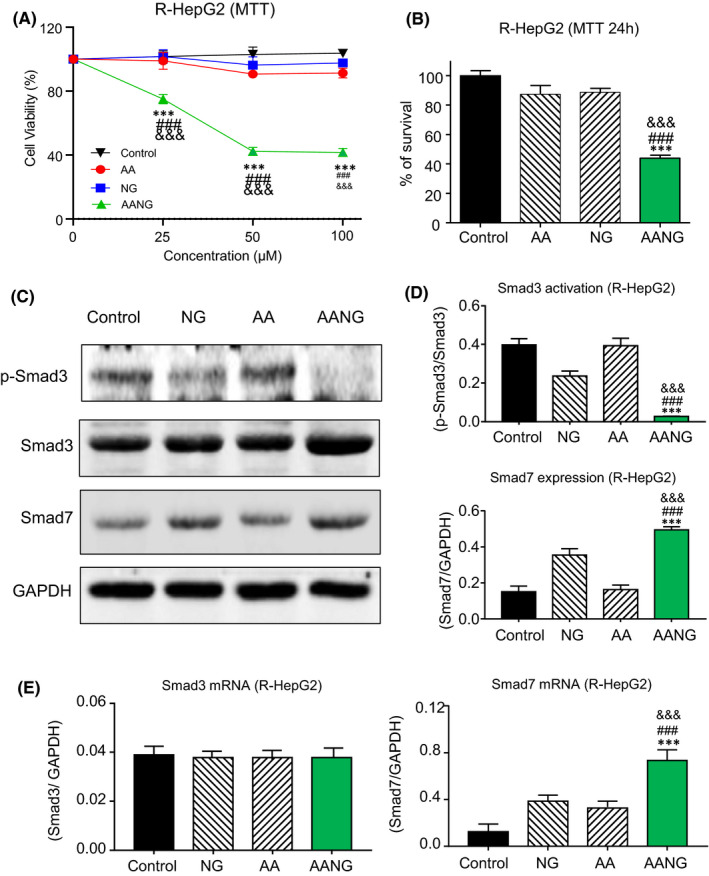
Combination of AANG inhibits multidrug resistant hepatocellular carcinoma (HCC) in vitro. (A) The inhibitory effect of AA, NG or their combination AANG on the proliferation of multidrug resistant human HCC cell line R‐HepG2 were detected by MTT assay at 24 h in vitro. (B) Interestingly, AANG (50 mM of AA +50 mM of NG) effectively suppressed the growth of R‐HepG2 cells at 24 h, whereas no significant effect was found in the groups treated with 50 mM of AA or NG in vitro. (C‐E) Western blotting and real‐time PCR showed that AANG synergistically suppressed Smad3 phosphorylation but triggered Smad7 expression in R‐HepG2 cell at 24 h, compared to the mono‐treatments with AA or NG. ***p < 0.001 vs. Control, ^###^ *p* < 0.001 vs. NG, ^&&&^ *p* < 0.001 vs. AA

According to our previous works, AANG works in a disease and cell type specific manner.^24^, ^34^ Therefore, we evaluate the optimal dose and ratio of AANG by using our unique multidrug resistant human HCC cell line R‐HepG2 with strong p‐glycoprotein expression.^31^ AA, NG and their combination in 1:1 ratio were applied on the R‐HepG2 cells with a dosage range (0 to 400 µM) for 24 and 48 h. Encouragingly, we found that combination of AA and NG synergistically inhibited the proliferation of R‐HepG2 cells with IC50 value at 50 µM in vitro, whereas 300 µM and 250 µM were needed for AA and NG respectively **(**Figure 2A and Figure S2). In contrast, there was no significant cytotoxicity detected in the groups received AA or NG alone compared to the solvent control (Figure 2B). To test the specificity of AANG on the Smad signalling, we treated the R‐HepG2 cells with AA, NG or their combination at 50 µM for 24 h and then submitted for Western blot analysis. As shown in Figure 2C‐E, AANG effectively altered both Smad3 and Smad7 in the R‐HepG2 cells in vitro, which cannot be achieved by using either AA or NG alone. To note, we found that AANG markedly increased the transcription of Smad7 but not Smad3 of in the R‐HepG2 cells in vitro, suggesting the inhibitory effect of AANG on Smad3 is mainly due to post‐transcriptional regulation (Figure 2E). These findings suggested a therapeutic potential of AANG for multidrug resistant HCC via rebalancing the Smad signalling in a synergistic manner.

### 3.3 AANG effectively blocks progression of R‐HepG2 xenografts in vivo

**FIGURE 3:**
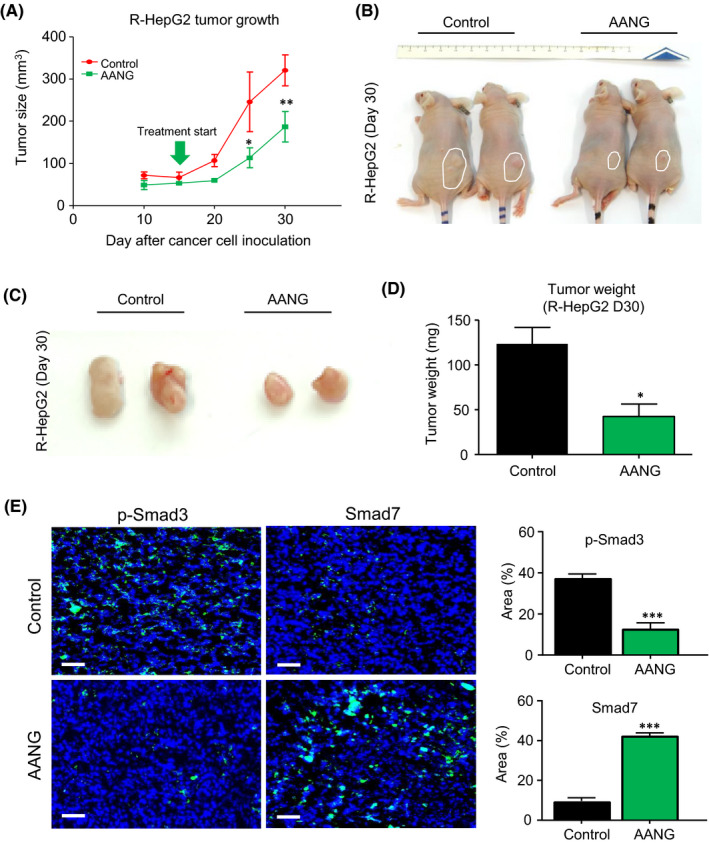
AANG effectively inhibits the progression of R‐HepG2 xenograft in vivo. (A‐D) Treatment with AANG significantly suppressed the growth of human hepatocellular carcinoma (HCC) xenograft R‐HepG2 in nude mice, showing by a significant reduction in the (A) tumour growth rate, (B‐C) size and (D) weight on day 30. (E) Additionally, AANG treatment effectively rebalanced the Smad signalling in the R‐HepG2 xenograft in vivo, showing by the markedly inactivation of Smad3 but up‐regulation of Smad7 in the treated mice compared to their control group. *p < 0.05, **p < 0.01, ***p < 0.001 vs. Control, *n* = 5, one‐way ANOVA. Scale bars (E) 50 μm

**FIGURE 4:**
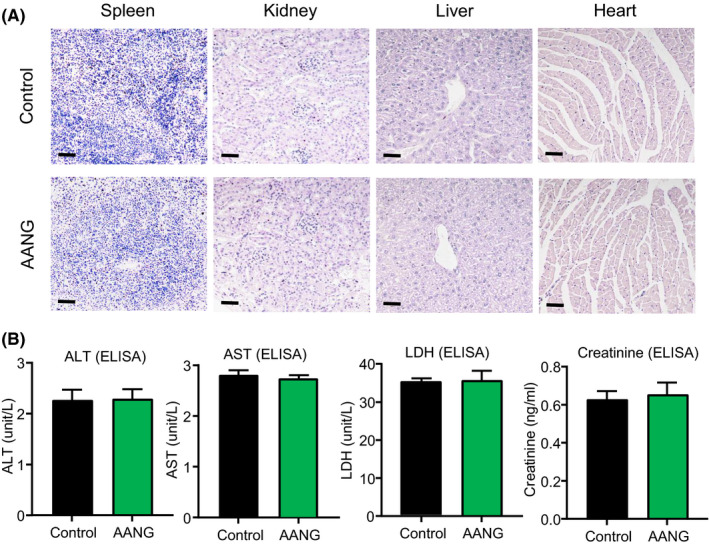
AANG is a safe anticancer therapy without significant side effects. The safety of AANG was evaluated with the serum and important organs collected from the R‐HepG2 xenograft bearing mice. Encouragingly, (A) no pathological damages were observed in the important organs of the AANG‐treated mice on Day 30 showing by H&E staining. (B) The safety of AANG was further confirmed by the insignificant changes of serum levels of ALT, AST, LDH and creatinine in the serum of the AANG‐treated mice, detecting by ELISA. Scale bars (A) 50 μm

Therefore, we further evaluated the therapeutic potential and safety of AANG in nude mice bearing the R‐HepG2 xenograft which representing the human multidrug resistant HCC. According to the growth curve of R‐HepG2 xenograft, our results demonstrated that AANG effectively suppressed the growth of R‐HepG2 xenograft on nude mice compared to the control group (Figure 3A,B). To note, the tumour size and weight were significantly reduced by AANG therapy at Day30 (Figure 3C,D). Furthermore, we confirmed that AANG effectively rebalanced the Smad signalling of the R‐HepG2 xenograft, showing by a significant inactivation of Smad3 but increment of Smad7 (Figure 3E). More importantly, AANG showed no significant side effects to the important organs of cancer host, confirming by histological observation on the spleen, kidney, liver and heart tissues (Figure 4A) and enzymatic analysis of the alanine aminotransaminase (ALT), aspartate aminotransaminase (AST), lactate dehydrogenase (LDH) and creatinine (Figure 4B).

### 3.4 AANG overcomes multidrug resistance by targeting a Smad3/MDR1 axis

**FIGURE 5:**
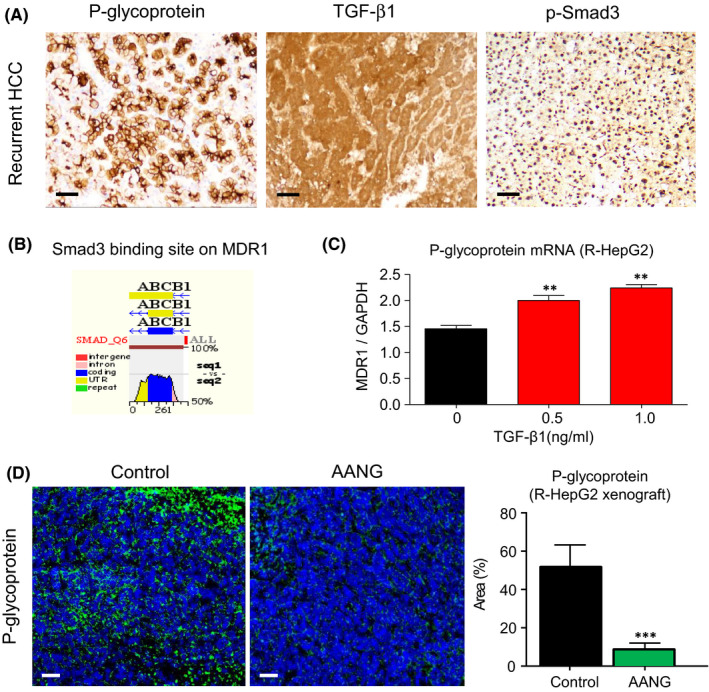
AANG overcomes p‐glycoprotein mediated multidrug resistance of hepatocellular carcinoma (HCC). (A) Expression of multidrug resistance mediator p‐glycoprotein was highly associated with the activation of TGF‐β1/Smad3 signalling in the recurrent HCC (*n* = 10). (B) A Smad3 binding site (red) at the 5′ UTR of p‐glycoprotein (gene name ABCB1 or MDR1) on the human and mouse evolutionarily conserved genomic region was detected by ECR browser bioinformatic platform. (C) Real‐time PCR shows that TGF‐β1 significantly triggers mRNA expression of p‐glycoprotein in R‐HepG2 cells in dose‐dependent manner in vitro. (D) More importantly, AANG therapy successfully cancelled p‐glycoprotein expression in the R‐HepG2 xenograft bearing mice compared to their control group in vivo. **p < 0.01, ***p < 0.01 vs. Control, *n* = 5, one‐way ANOVA. Scale bars (D), 50 μm

Multidrug resistance is an unsolved clinical problem of HCC, where ATP‐binding cassette (ABC) transporter p‐glycoprotein is one of the well‐documented therapeutic targets.^35^ We observed that overexpression of p‐glycoprotein was closely associated with the activation of TGF‐β1/Smad3 signalling in the recurrent HCC (Figure 5A). As shown in Figure 5B, by conducting bioinformatic analysis with ECR browser as our previous studies,^15^, ^16^, ^20^ we revealed a direct Smad3 binding site on the 5′ untranslated region of p‐glycoprotein genomic sequence (gene name MDR1 or ABCB1). In addition, we demonstrated that transcription of MDR1 was significantly increased in the R‐HepG2 cells by TGF‐β1 stimulation in a dose‐dependent manner (Figure 5C), implying a regulatory role of TGF‐β1/Smad3 signalling in p‐glycoprotein expression at transcriptional level. More importantly, we found that AANG therapy dramatically suppressed p‐glycoprotein expression in the R‐HepG2 xenograft compared to the control group in vivo (Figure 5D). In addition, AANG effectively converted the protumoural TME into anticancer showing by a marked reduction in angiogenesis but increment of NK cells in the R‐HepG2 xenografts of treated mice (Figure S3). Thus, AANG may represent a novel, effective and safe TCM‐based natural compound formula for HCC especially with p‐glycoprotein mediated multidrug resistance.

## 4 DISCUSSION

Multidrug resistance is an important barrier in cancer especially HCC due to the detoxification characteristic of its origin organ liver.^35^ Increasing evidence suggested a regulatory role of TGF‐β1/Smad signalling in the p‐glycoprotein mediated multidrug resistance^11^, ^12^ and the tumour microenvironment driven cancer progression.^5^, ^21^ We have developed a natural compound formula AANG for correcting the imbalance of TGF‐β1/Smad signalling under tissue inflammation.^24^, ^34^ This is the first study to explore the therapeutic potential of AANG for multidrug resistant HCC. We evidenced the imbalance of Smad3 and Smad7 equilibrium in HCC patient biopsies, which was markedly enhanced in the recurrent cases associated with the p‐glycoprotein expression. We optimized the ratio and dosage of AANG by using a well‐documented multidrug resistant human HCC cell line R‐HepG2.^31^, ^36^ Encouragingly, the optimal AANG formula effectively inhibited the growth of R‐HepG2 xenograft and their p‐glycoprotein expression in the nude mice. Importantly, no side effect was detected in the R‐HepG2 xenograft bearing mice after AANG therapy. Thus, AANG is a novel, specific, effective and highly safe TCM‐based natural compound formula for human cancer even with multidrug resistance.

Cancer is still a leading cause of death worldwide due to its lack of effective treatment especially for the recurrent cases. TME is a new therapeutic target for cancer due to its importance for promoting cancer growth, invasion, metastasis and drug resistance.^1^, ^2^ Recently, we discovered the essentialness of Smad3 in the microenvironment for developing inflammatory diseases including cancer.^1^, ^5^, ^13^, ^21^ Smad3 deficiency protected mice against tumour growth, invasion and metastasis, where a markedly reduction in angiogenesis (CD31, VEGF), invasion and metastasis (MMPs), and immunosuppression (decrease in Foxp3^+^ Treg but increase in NKp46^+^ NK cells) were found in the Smad3‐KO mice compared to the wildtype mice, revealing the important role of Smad3 signalling in the protumoural TME.^21^ Encouragingly, in this study, we also detected that AANG markedly suppressed our reported Smad3‐mediated NK immunity suppression in the R‐HepG2 xenografts in vivo,^21^ thereby blocking the TME‐driven cancer development and progression in mice.

In fact, Smad7 suppression is associated with and resulted in Smad3 activation in inflammatory diseases including cancer.^37^ Besides targeting Smad3, overexpression of Smad7 also effectively inhibit cancer in animal models.^38^ Smad7 is a negative regulator of TGF‐β/Smad3 signalling pathway.^39^ Despite targeting Smad3‐TME,^15^ overexpression of Smad7 also inhibits primary tumour growth and metastasis in a number of cancer models.^24^, ^38^ Dramatic reduction in Smad7 but activation of Smad3 occurs in many inflammatory disease conditions including cancer.^17^, ^19^ Based on these evidence‐based observations, Smad3 may play a pathogenic promoter in cancer, whereas Smad7 may be protective. Thus, correcting the equilibrium of Smad3/Smad7 in the TME may produce a synergetic anticancer outcome. Indeed, our data also showed that AANG synergistically increased the expression of Smad7 in R‐HepG2 at transcriptional level and in the TME of R‐HepG2 xenografts in vivo. AANG may represent an ideal therapeutic strategy for cancers showing unbalanced Smad signalling in the TME.

TGF‐β1 is crucial for the development of TME, which largely promotes regulatory T‐cell infiltration, angiogenesis, epithelial‐mesenchymal transition (EMT) etc.^3^, ^4^ Unexpectedly, TGF‐β1 also triggers the expression of multidrug resistance p‐glycoprotein expression in blood‐brain barrier and cancer cells.^11^, ^12^ Thus, targeting of TGF‐β receptors with soluble TGF‐β receptor II, small molecule ALK5 kinase inhibitors, or neutralizing TGF‐β1 antibodies have been reported to exhibit some therapeutic effect on cancer.^2^ However, as TGF‐β1 is a fundamental anti‐inflammatory cytokine and immune regulator, blockade of TGF‐β1 since receptor level will cause autoimmune diseases. In this study, high level of TGF‐β1 was observed in the biopsies of cancer patients with hepatoma, which was associated with Smad3 hyperactivation but Smad7 reduction in the TME. Therefore, identification of a more specific therapeutic for targeting in the downstream of TGF‐β1 signalling is in urgent need. AANG caused no damage to the main organs of the cancer host including heart, liver, spleen and kidney. Besides, both AA and NG have several clinical studies such as using NG in hypercholesterolaemic and overweight human subjects and using AA in patients with Alzheimer's disease, both AA and NG show no adverse effects in the studies^40^, ^41^, ^42^; highlighting its translational potential for targeting the TGF‐β1 signalling in cancer.

We have recently reported that AA is a Smad7 agonist and NG is a Smad3 inhibitor in lung carcinoma.^24^ However, AA and NG using alone could not effectively regulate the Smad7 and Smad3 expression in HCC. Interestingly, our data revealed that AANG can synergistically trigger Smad7 expression in the R‐HepG2 cells compared to the monotherapy with AA or NG only, resulting in a better anticancer effect in vitro. To note, Smad7 is a negative regulator of NF‐κB signalling pathway,^1^, ^5^, ^17^, ^39^ which NF‐κB is highly activated in cancer and regulates the p‐glycoprotein expression in HCC.^43^ Our experiment showed that AANG effectively inhibits the cancer progression of R‐HepG2 bearing mice associated with a dramatic reduction of p‐glycoprotein in the HCC xenografts. This encouraging data revealed the therapeutic potential of AANG for blocking the NF‐κB‐driven p‐glycoprotein mediated multidrug resistance via upregulating Smad7 in a synergistic manner.

To conclude, it is the first study systematically evidenced the imbalance of Smad signalling in the TME of HCC especially in the recurrent cases with high p‐glycoprotein expression level. We successfully optimized our TCM‐derived natural compound formula AANG for the multidrug resistant HCC, which specifically inhibited Smad3 activation but up‐regulated Smad7 expression in both cancer cells and the TME in vitro and in vivo. Importantly, AANG effectively blocked the progression of multidrug resistant HCC without detectable side effects in mice. Thus, AANG may represent a novel, safe and effective therapeutic strategy for HCC with p‐glycoprotein mediated multidrug resistance.

## CONFLICT OF INTEREST

The authors declare that there are no competing interests.

## AUTHOR CONTRIBUTIONS

**Jeff Yat‐Fai Chung:** Data curation (lead); Investigation (lead); Methodology (lead); Visualization (lead); Writing‐original draft (equal). **Max Kam‐Kwan Chan:** Data curation (equal); Formal analysis (equal); Investigation (equal); Writing‐review & editing (lead). **Philip Chiu‐Tsun Tang:** Methodology (supporting); Validation (supporting); Writing‐review & editing (supporting). **Alex Siu‐Wing Chan:** Writing‐review & editing (equal). **Justin Shing‐Yin Chung:** Methodology (equal); Validation (equal). **Xiao‐Ming Meng:** Validation (supporting); Visualization (supporting). **Ka‐Fai To:** Resources (supporting); Writing‐review & editing (supporting). **Hui‐Yao Lan:** Resources (equal); Supervision (supporting); Validation (equal). **Kam‐Tong Leung:** Funding acquisition (equal); Supervision (equal); Writing‐review & editing (equal). **Patrick Ming‐Kuen Tang:** Conceptualization (lead); Funding acquisition (lead); Investigation (lead); Supervision (lead); Writing‐original draft (lead); Writing‐review & editing (lead).

## Supporting information

Supplementary Material

## Data Availability

The data that support the findings of this study are available from the corresponding author upon reasonable request.
