# Supplementary material for: AANG: A natural compound formula for overcoming multidrug resistance via synergistic rebalancing the TGF‐β/Smad signalling in hepatocellular carcinoma
Source: J Cell Mol Med. 2021 Sep 12;25(20):9805–13. doi: 10.1111/jcmm.16928 (PMC8505848; doi:10.1111/jcmm.16928)
Supplement: Supplementary file 1 — Supplementary Material [file JCMM-25-9805-s001.pdf]

Western Blotting		
**Primary Antibodies**	**Catalogue Number**	**Manufacturer**
p-SMAD3	sc-517575	Santa Cruz
SMAD3	sc-101154	Santa Cruz
SMAD7	sc-365846	Santa Cruz
GAPDH	sc-32233	Santa Cruz
**Histology and Immunofluorescence Staining**		
**Primary Antibodies**	**Catalogue Number**	**Manufacturer**
MDR1	sc-55510	Santa Cruz
p-SMAD3	sc-517575	Santa Cruz
TGF-β1	sc-130348	Santa Cruz
SMAD7	sc-365846	Santa Cruz

Supplementary Table S1. List of antibodies used in this study.

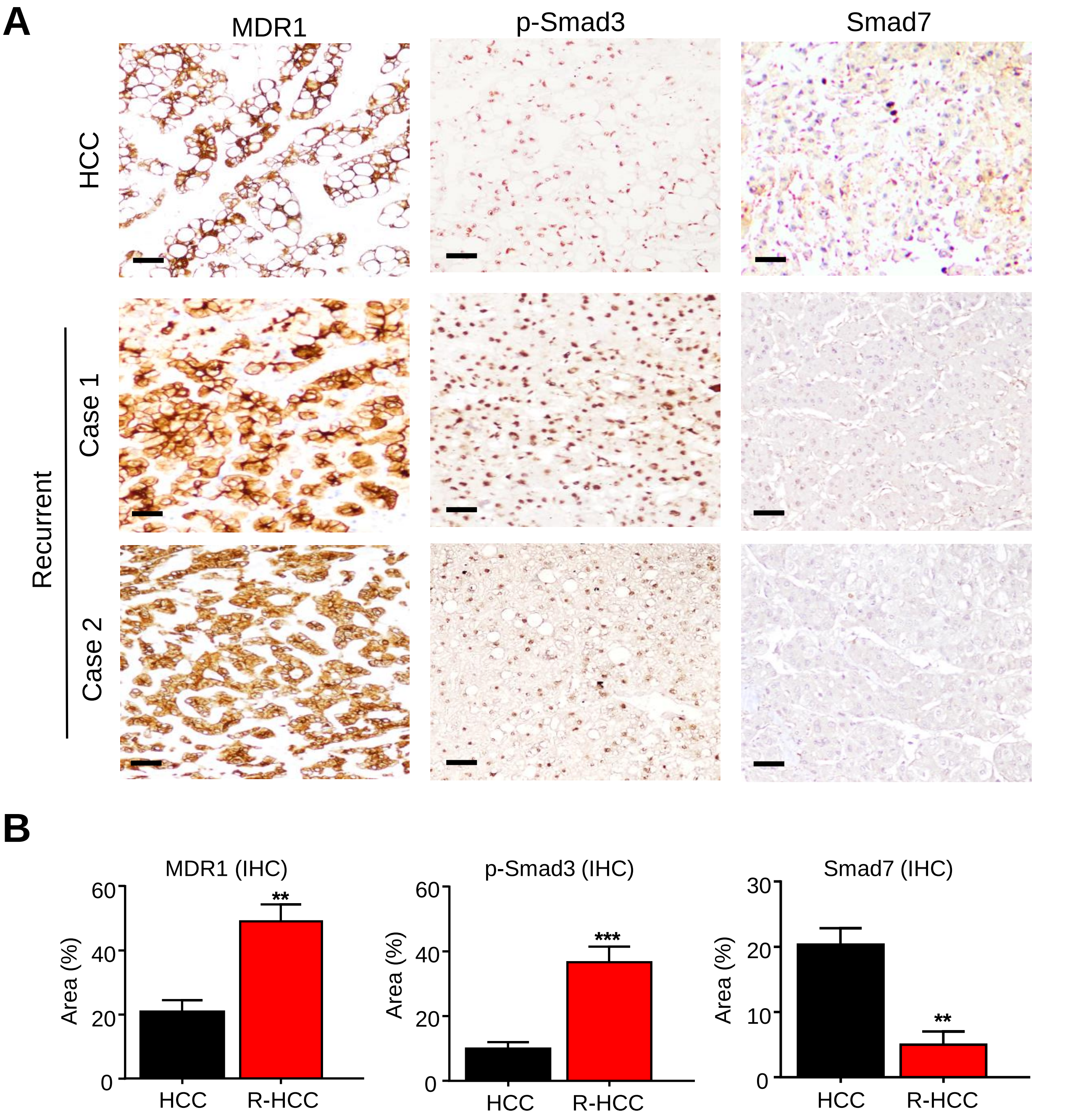

Supplementary Figure S1. Imbalance of the Smad singaling in patients with primary and recurrent HCC. The imbalance of Smad3 and Smad7 significantly associates with upregulated p-glycoprotein level in both the primary and recurrent HCC, detected by **(A)** Immunohistochemistry and (**B**) their quantification. **P <0.01, ***P <0.01 vs HCC, n=6, one-way ANOVA. Note: The upper two IHC samples are originated from Figure 1, and the lowest panel is an additional case of recurrent HCC. Scale bar (**A**), 50 μm.

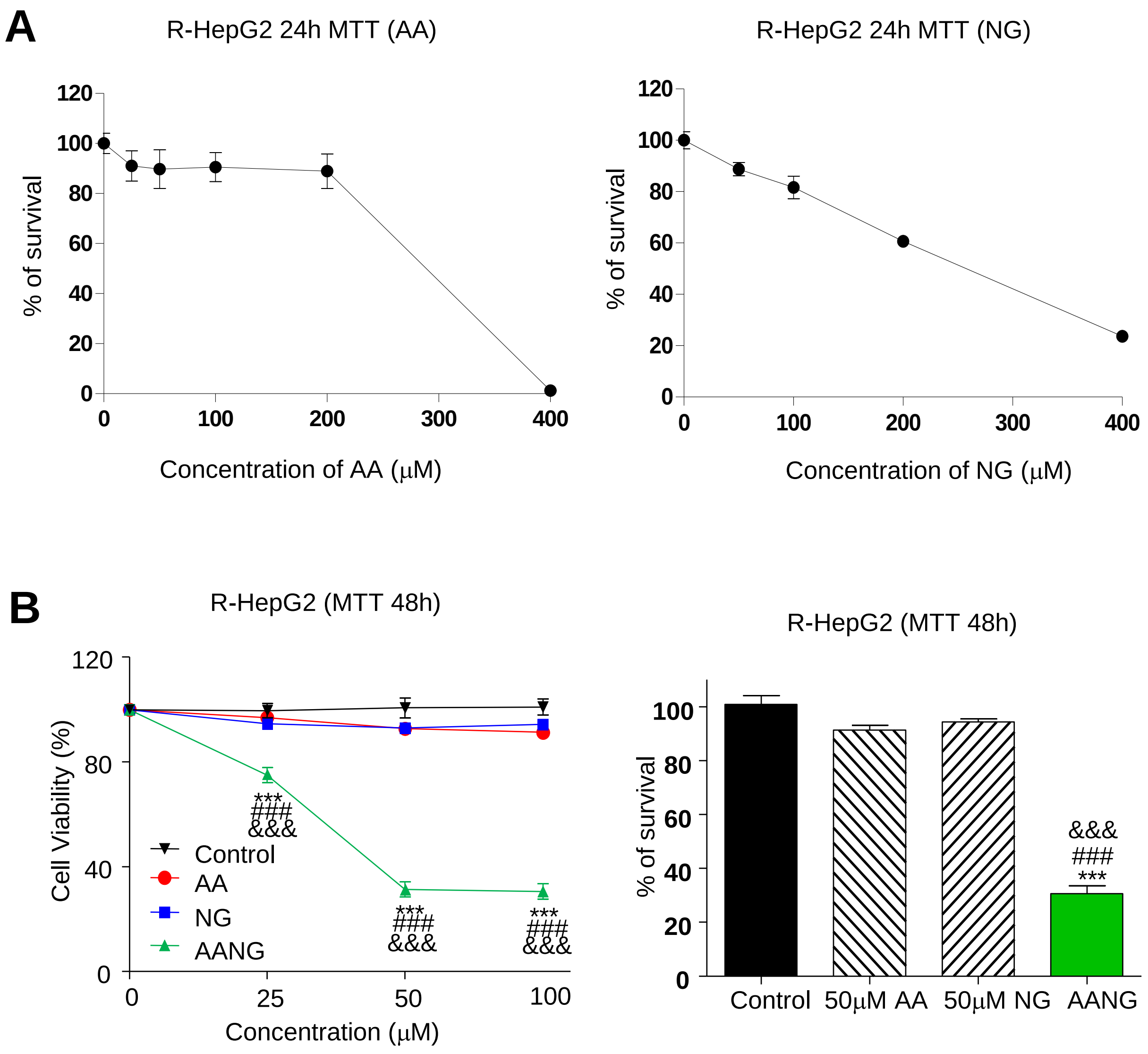

Supplementary Figure S2. Effect of AA or NG on the proliferation of R-HepG2 cells *in vitro*. Higher IC50 of (**A**) AA or NG monotreatment compared to (Figure 2A) combined treatment were detected by MTT assay on the R-HepG2 cells with 24h treatment *in vitro*. **(B)** Synergistic inhibitory effect on R-HepG2 growth of AA and NG combination since 25 μM was also observed in 48h treatment *in vitro*. ***P <0.001 vs Control, $^{###}$P <0.001 vs NG, $^{\&\&\&}$P <0.001 vs AA.

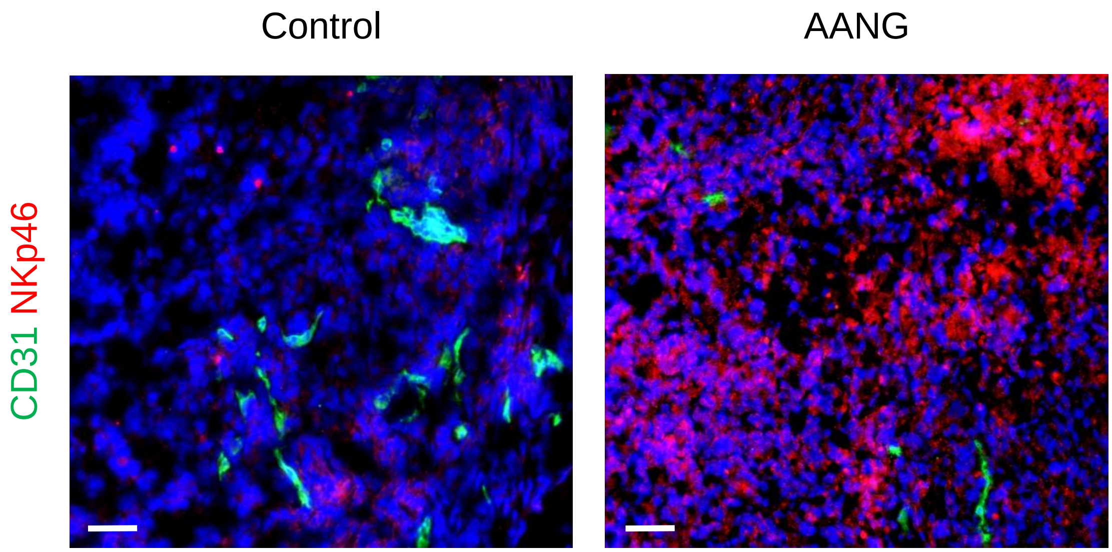

Supplementary Figure S3. AANG effectively suppressed the protumoral microenvironment of R-HepG2 *in vivo*. AANG markedly inhibited angiogenesis (CD31) but promoted anticancer natural killer cells (NKp46) in R-HepG2 xenografts *in vivo* (n=8). 50 μm.
